# Changes in receptivity epithelial cell markers of endometrium after ovarian stimulation treatments: its role during implantation window

**DOI:** 10.1186/s12978-015-0034-7

**Published:** 2015-05-17

**Authors:** Francisco J. Valdez-Morales, Armando Gamboa-Domínguez, Victor S. Vital-Reyes, Juan C. Hinojosa Cruz, Jesús Chimal-Monroy, Yanira Franco-Murillo, Marco Cerbón

**Affiliations:** Unidad de Investigación en Reproducción Humana, Instituto Nacional de Perinatología-Facultad de Química, Universidad Nacional Autónoma de México, México, D.F. Mexico; Departamento de Patología, Instituto Nacional de Ciencias Médicas y Nutrición Salvador Zubirán, México, D.F. Mexico; Departamento de Biología de la Reproducción, Hospital de Ginecología y Obstetricia # 3, Centro Médico Nacional La Raza, Instituto Mexicano del Seguro Social, México, D.F. Mexico; Departamento de Genómica y Toxicología Ambiental, Instituto de Investigaciones Biomédicas, UNAM, México, D.F. Mexico; Instituto Nacional de Perinatología, México, D.F. Mexico

**Keywords:** Infertility, Human endometrium, Ovulation induction, Clomiphene citrate, Recombinant follicle stimulating hormone, Receptivity markers

## Abstract

**Background:**

To compare the expression of receptivity markers in epithelial and stromal cells in the endometrium of ovulatory women and infertile with hypothalamic pituitary dysfunction (HPD), untreated or treated with clomiphene citrate (CC), or with recombinant follicle stimulating hormone (rFSH).

**Methods:**

Twelve control ovulatory and 32 anovulatory women, 22 of whom received ovulation induction with CC (n = 12) or rFSH (n = 10). Endometrial biopsies were obtained during the mid-secretory phase. Hormonal secretion was measured by chemiluminescence immunoassay, endometrial dating and cellular expression and distribution of receptivity proteins were evaluated by quantitative immunohistochemistry.

**Results:**

CC or rFSH treatments, modified the expression of epithelial receptivity markers, such as Glycodelin A, beta-catenin, CD166/ALCAM and IGF-1R, but not in stromal markers. Also, a change in their cell distribution was observed.

**Conclusions:**

Treatment of infertile women with HPD modified the expression and distribution of receptivity markers in the mid-secretory phase of the endometrium in epithelial but not stromal cells, which can help to explain changes in the receptivity of the endometrium during treatments and suggest an important role of these cells in the receptivity window.

**Electronic supplementary material:**

The online version of this article (doi:10.1186/s12978-015-0034-7) contains supplementary material, which is available to authorized users.

## Background

Biochemical and morphological changes of the endometrium during the secretory phase of the menstrual cycle depend on complex cell signaling that leads to the receptive state for embryo implantation. Consequently, alterations of molecular or cellular interactions during this period could have a negative impact on the success of pregnancy [1,2].

Human endometrial receptivity refers to the ability of the uterus to accept and develop a new embryo [3]. Several studies have identified cell signaling pathways and specific proteins involved in maturation, differentiation and functionality of the endometrium [4,5]. Among proteins expressed in luminal and glandular epithelia are: cadherins, β-catenin, CD166/ALCAM, glycodelin A (GdA), leukemia inhibitory factor (LIF), stem cell factor (SCF) and its receptor (c-Kit), epithelial growth factor (EGF), mucin-1 (MUC1), integrin αVβ3 and the insulin like growth factor (IGF) family [6–8]. Moreover, stromal protein changes induced by cytokines and other immune mediators, including interleukins (IL)-6 and 11, vascular endothelial growth factor (VEGF), transforming growth factor beta (TGF-β) family, CD34, CD31/PCAM-1, CD44, matrix metalloproteinase proteins (MMP) and the transcriptional regulators FOXO1, and HOXA10 [7,9] affect endometrial vascularization and decidualization at varying degrees [10]. We and others have found that infertile patients undergoing assisted reproduction or ovarian stimulation protocols develop molecular alterations of the endometrium [11–15].

Hypotalamic pituitary dysfunction (HPD) is characterized by lutheal phase deficiency, oligomenorrhoea and anovulation with normal basal levels of serum FSH, LH and estradiol [16,17]. The first election treatment involves ovulation induction with clomiphene citrate (CC) or gonadotrophins such as recombinant FSH (rFSH) to obtain no more than two mature follicles. However the side effects of the treatments include a decrease in endometrial thickness and quality and also a reduction of the number of glands per mm2 with smaller gland diameter during secretory phase in CC-treated women, whereas rFSH treatment increase the endometrial thickness [18,19]. At molecular level, previously we reported an alteration of proliferation and cell death markers expression as well as hormonal receptors in both treatments [15]. On the other hand, it has also been shown that only 30 % of all stimulated patients with CC or rFSH becomes pregnant in spite of the efficient restitution of ovulation [15,18]. In the present work we examined the molecular effects of ovulation inducing agents (CC or rFSH), on endometrial expression of stromal and epithelial receptivity markers mentioned above and their cell distribution in the mid-secretory endometrium of women with HPD.

## Methods

### Tissue collection

The study included women in reproductive age (26–31 years old) that either had normal ovulation or were anovulatory and infertile. The latter category included women with a diagnosis of HPD (according to the WHO criteria, Type II infertility) [17] or oligomenorrhea. Women participating in this study were patients at the National Medical Center “La Raza” of the Instituto Mexicano del Seguro Social from January, 2009, to March, 2011. All patients gave their informed consent, and the study was approved by the Institutional and National Mexican Ethics Committees (trial registration number: R-2014-3504-8) and performed in accordance with international laws, according to Declaration of Helsinki on procedures for the handling of human tissue.

### Clinical treatment of the patients

Endometrial samples were obtained and divided into four groups: a) control ovulatory women (n = 12); b) anovulatory infertile patients treated with 100 mg of CC per day from days 5 to 9 of the cycle (n = 12); c) anovulatory infertile patients treated with a step-up schedule of rFSH (n = 10); d) anovulatory infertile patients with oligomenorrhea and without treatment (n = 10). The patients without previous fertility treatments were randomized for the administration ovarian stimulation treatments. The rFSH treatment started on day 3 of the menstrual cycle, with a dose of 50–75 IU per day. Follicular growth was evaluated by ultrasonography on days 9–10, and in accordance with this parameter doses were either maintained or increased (in the latter case, up to 100–150 IU of rFSH). Follicular growth was re-evaluated on day 11–12 of the cycle to determine if a follicular diameter of 18 mm had been attained. In such a case, doses were maintained. If not, the cycle was canceled.

Treated patients (groups’ b and c) were given 2 mg of chlormadinone for 10 days. After menstruation, patients were treated with CC or rFSH on the indicated days. All women underwent an endometrial biopsy with Pipelle cannula (CCD International, Paris, France) while they were in the mid-secretory phase of the menstrual cycle (LH + 7 days), which was determined based on the last menstrual period and confirmed by histological dating. All patients were monitored during treatments with echographic endometrial pattern and thickness evaluation, and of the growth and number of follicles. Hysterosalpingography was carried out for diagnosis and to exclude other causes of infertility. Biopsies of untreated infertile women with oligomenorrhea were obtained randomly.

### Endometrial histology and dating

Endometrial tissues were fixed in 3 % formalin and left for 24 h at room temperature and we performed the hematoxilin eosin staining as we reported before [15]. The sections were observed in a Nikon Eclipse E600 light microscope for analysis and photographs were taken. The endometrial tissue was examined, dated and analyzed histologically according to Noyes criteria [20].

### Hormonal measurement

The hormonal serum levels of FSH, LH, estradiol, progesterone and prolactin (PRL) were determined in duplicate by chemiluminescence immunoassay (Inmulite kit Diagnostic Products Corporation, Los Angeles, CA, USA) before treatment (basal concentrations) and after treatment on the day of the biopsy. The intra- and inter-assay coefficients of variation were 5.4 and 6.6 % for FSH, 6.5 and 10 % for LH, and 4.4 and 5.4 % for estradiol, respectively.

### Immunohistochemistry

As we reported previously [15], tissues were processed and embedded in paraffin, and used for immunohistochemistry assay to evaluate the expression of the epithelial receptivity markers: GdA (Santa Cruz, CA, Dallas, TX, USA; 1:100), LIF (R&D Systems, Minneapolis, MN, USA; 1:100), C-Kit (Biocare, Concord, CA, USA; 1:250), SCF (Biocare, Concord, CA, USA; 1:100), E-cadherin (Dako, Carpinteria, CA, USA; 1:50), beta-catenin (Biocare, Concord, CA, USA; 1:50) and IGF-1R (Biocare, Concord, CA, USA; 1:100). To evaluate molecules related to endometrial stromal decidualization, we used: ALCAM/CD166 (Abcam, San Francisco, CA, USA; 1:100), TGF-β (Santa Cruz, CA, Dallas, TX, USA; 1:100), VEGF (Biocare, Concord, CA, USA; 1:100), CD34 (Biocare, Concord, CA, USA; 1:100), CD44 (Biocare, Concord, CA, USA; 1:100) and CD31/PCAM-1 (Biocare, Concord, CA, USA; 1:100). Tissues were analyzed in a Nikon Eclipse E600 microscope and photographs from three different tissue areas were taken randomly.

### Image processing and analysis

The staining intensity (mean gray value) of immunopositive cells was evaluated with NIH Image J software. The gray level was converted to a numerical value using a scale of 0 (white) to 255 (black), as described previously [21]. The immunopositive cell index was obtained by the ratio of positive cells to total cells.

### Statistical analysis

All data presented a Gaussian distribution, as shown by analysis with D'Agostino & Pearson omnibus normality test (Prisma 5.0 GraphPadSoftware, San Diego, CA, USA). Immunohistochemistry data were analyzed by using one-way ANOVA on each data set followed by the *post hoc* Bonferroni test for multiple comparisons. The Prism 5.0 program (Graph Pad Software, San Diego, CA, USA) was used for calculating probability values. Results were considered statistically significant with *p* < 0.05.

## Results

1. Histological endometrial dating and hormonal levels

In concordance with our previous reported observations, in patients with HPD, we did not find histomorphological differences between normal and treated women [15]. In addition, the histological dating of the endometrium demonstrated similar values of endometrial development in all groups. Moreover, evaluation of hormonal levels of PRL, E_2_, P_4_, LH and FSH indicated normal values in all groups (Table 1).

**Table 1:** Summary of clinical data from patients included in all groups

	OP	UI	CC-treated	rFSH-treated
No. of patients (n)	12	10	12	10
Age (years)	30.83 ± 3.27	31.32 ± 3.58	31 ± 4.39	30.46 ± 3.95
Body mass index (kg/m^2^)	26.24 ± 2.70	26.48 ± 2.91	26.74 ± 2.80	25.98 ± 2.70
Basal hormonal profile				
PRL (ng/mL)	17.46 ± 2.44	13.78 ± 2.32	15.93 ± 2.82	12.75 ± 3.11
E_2_ (pg/mL)	35.13 ± 1.85	27.34 ± 3.12	46.61 ± 2.67	24.8 ± 2.28
LH (IU/L)	6.42 ± 1.16	4.98 ± 0.84	4.82 ± 1.07	5.80 ± 0.77
FSH (IU/L)	7.27 ± 0.94	8.86 ± 1.16	5.79 ± 0.79	6.07 ± 0.64
LH + 7 day hormonal profile				
E_2_ (pg/mL)	26 ± 2.04	31.05 ± 3.08	25.33 ± 2.16	48.51 ± 3.27
P_4_ (ng/mL)	12.01 ± 1.54	7.19 ± 1.98	20.87 ± 4.32	6.97 ± 1.14
Histological dating (cycle day)	19 ± 0.42	19 ± 0.7	19 ± 0.44	17 ± 0.79

Clinical data from the ovulatory patients (OP), untreated infertile (UI), clomiphene citrate (CC) and recombinant follicle stimulating hormone (rFSH) treated patients. Data represents mean ± SE. Hormonal measurement at basal condition (day 3) and after treatments in LH + 7 day, as well as histological dating in all groups

2) Differential expression of epithelial glycoproteins

**Fig. 1:**
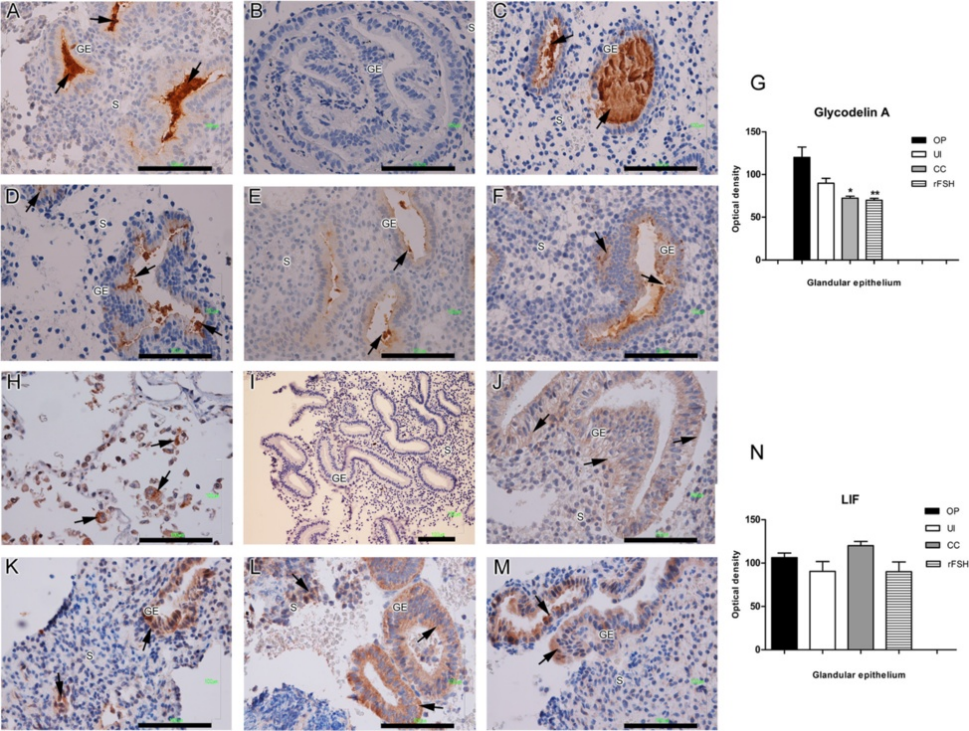
Endometrial expression of epithelial receptivity markers. Immunohistochemistry for GdA (**a** to **g**) and LIF (**h** to **n**). Positive staining was observed in glandular epithelium (brown and arrows), which was counterstained with Hematoxylin (blue nuclei). (**a**) Human mid-secretory endometrium and (**h**) human lung tissue was used as the positive control. (**b** and **i**) Human endometrium with no primary antibody served as the negative control. (**c** and **j**) Ovulatory women. (**d** and **k**) untreated anovulatory infertile women. (**e** and **l**) CC-treated anovulatory infertile women. (**f** and **m**) rFSH-treated women. (**g**) Optical density analysis of GdA in the glandular epithelium showed a significantly lower expression (*P < 0.05) in CC- and rFSH-treated groups compared with ovulatory and untreated infertile women. (**n**) Optical density analysis for LIF showed no significant differences between groups. GE = glandular epithelium; LE = luminal epithelium; S = stroma; OP = ovulatory women; CC = anovulatory infertile patients treated with clomiphene citrate; rFSH = anovulatory infertile patients treated with the recombinant follicle stimulating hormone; UI = anovulatory untreated patients. (Bar = 100 μm)

Total expression of GdA showed a diminution in treated patients in both CC and rFSH groups as compared with control and untreated infertile groups (Fig. 1). In contrast, no significant differences between groups were found with LIF expression (Fig. 1).

**Fig. 2:**
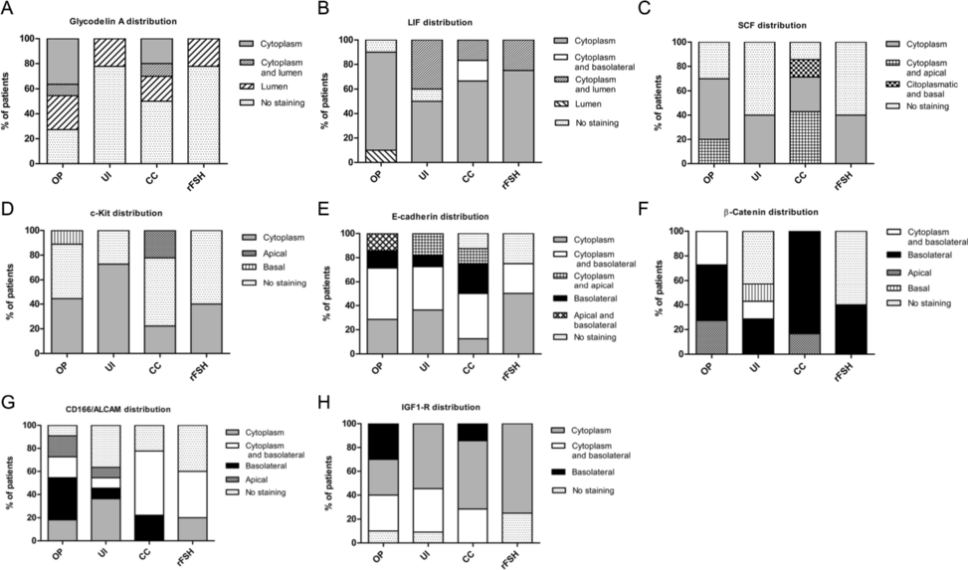
Epithelial protein distribution analysis. Percentage of patients that expressed a different cell location of epithelial receptivity markers in ovulatory patients (OP), untreated infertile (UI), CC treated and rFSH treated patients. (**a**) GdA, (**b**) LIF, (**c**) SCF, (**d**) c-Kit, (**e**) E-cadherin, (**f**) beta-catenin, (**g**) CD166/ALCAM, and (**h**) IGF-1R

**Fig. 3:**
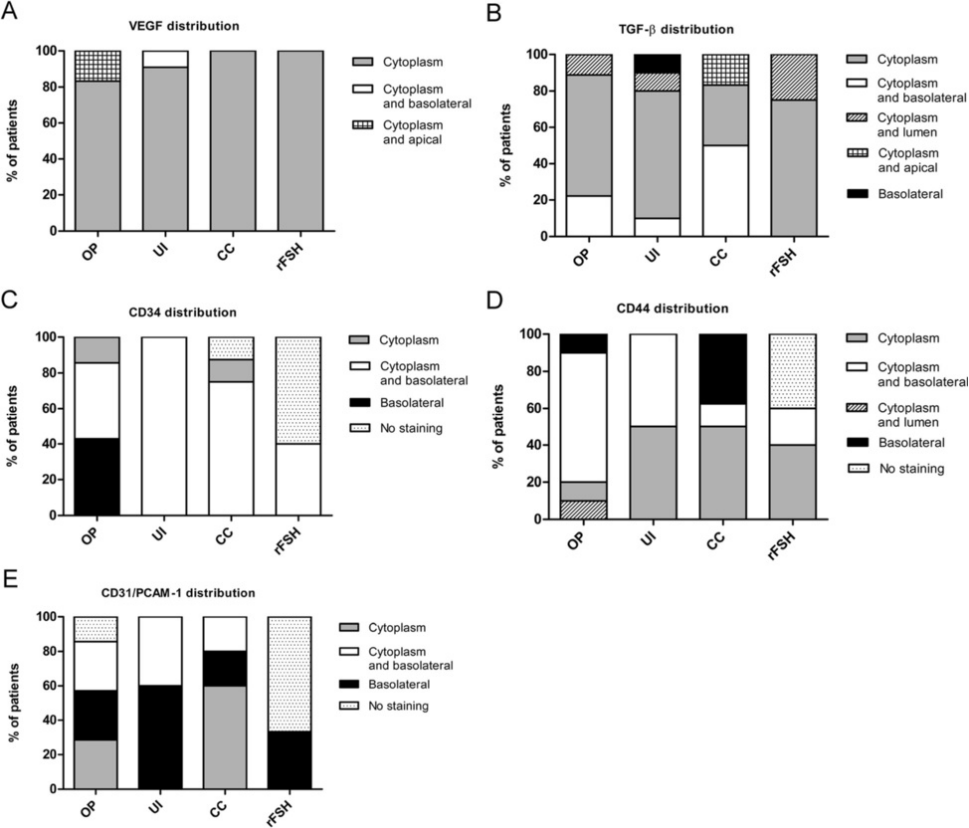
Stromal protein distribution analysis. Percentage of patients that expressed a different cell location of stromal receptivity markers in ovulatory patients (OP), untreated infertile (UI), CC treated and rFSH treated patients. (**a**) VEGF, (**b**) TGF-β, (**c**) CD34, (**d**) CD44 and (**e**) CD31/PCAM-1

Interestingly for GdA and all the studied markers a broad cellular distribution was observed and due to their complexity, these results were summarized in the Figs. 2 and 3. We found that GdA was expressed and distributed in the cytoplasm of glandular epithelial cells in 36 % of the ovulatory women. However, few if any staining was observed in the infertile groups. Nonetheless the cellular distribution pattern of GdA was similar to ovulatory women in CC treated group while in rFSH group the cellular distribution was similar with untreated infertile women (Fig. 2a). LIF expression was distributed mostly in the cytoplasm in all groups (Fig. 2b).

SCF and c-Kit immunostaining was observed in the cytoplasm of the glandular epithelial cells and also in the cell membrane of the stromal cells. However no significant differences were observed between groups (Additional file 1: Figure S1). The cellular distribution variations of these markers are shown in Fig. 2c and d.

3) Differential expression of epithelial cell adhesion molecules

**Fig. 4:**
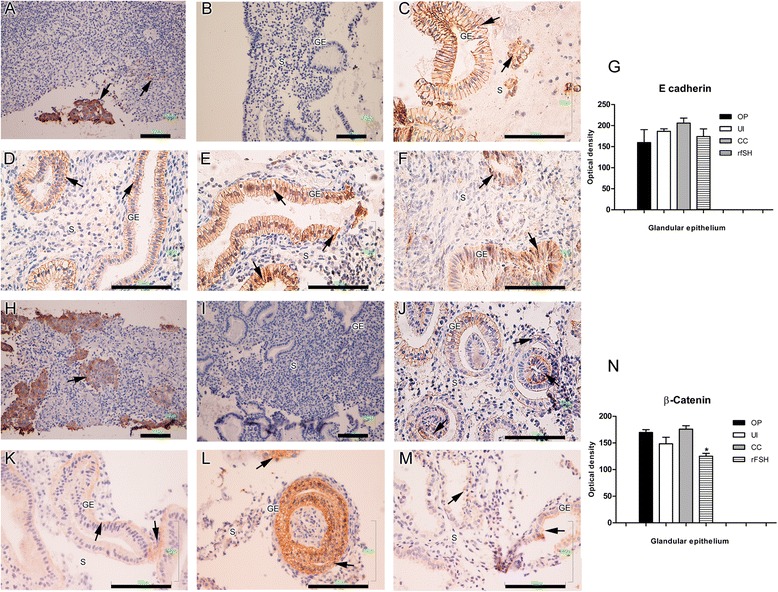
Endometrial expression of epithelial receptivity markers. Immunohistochemistry for E-cadherin (**a** to **g**) and β-catenin (**h** to **n**). Positive staining was observed in glandular epithelium and stroma (brown and arrows), which was counterstained with hematoxylin (blue nuclei). (**a** and **h**) Human skin cancer tissue was used as the positive control. (**b** and **i**) Human endometrium with no primary antibody served as the negative control. (**c** and **j**) Ovulatory women. (**b** and **k**) untreated anovulatory infertile women. (**e** and **l**) CC-treated anovulatory infertile women. (**f** and **m**) rFSH-treated women. (**g**) Optical density analysis of E-cadherin in the glandular epithelium and stroma showed no significant differences in expression between groups. (**n**) Optical density analysis for β-catenin showed a significantly lower expression (*P < 0.05) in the glandular epithelium of the rFSH-treated group compared with ovulatory and CC-treated women. GE = glandular epithelium; LE = luminal epithelium; S = stroma; OP = ovulatory women; CC = anovulatory infertile patients treated with clomiphene citrate; rFSH = anovulatory infertile patients treated with the recombinant follicle stimulating hormone; UI = anovulatory untreated patients. (Bar = 100 μm)

The expression of E-cadherin and beta-catenin staining was detected at epithelial cytoplasmic/membrane localization. Beta-catenin expression in the rFSH-treated group was significantly lower than those observed in ovulatory and CC-treated women. No significant differences were found in E-cadherin expression between groups (Fig. 4).

Beta-catenin was mainly distributed in the basolateral membrane in patients from the ovulatory group, and similar distribution was observed in CC-treated women. In contrast, no staining was predominantly observed in untreated infertile and rFSH treated groups. In the case of E-cadherin the cell distribution in ovulatory and CC-treated patients was observed in the cytoplasm and basolateral membrane, whereas the major distribution was located only in the cytoplasm of untreated infertile and rFSH-treated women (Fig. 2 e and f).

**Fig. 5:**
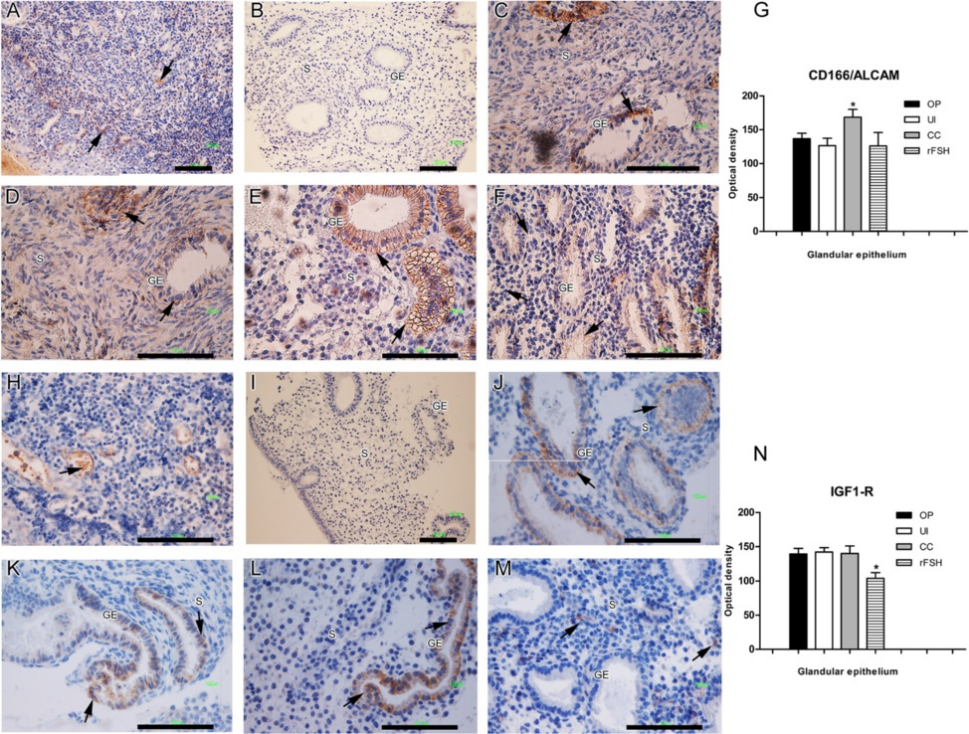
Endometrial expression of epithelial receptivity markers. Immunohistochemistry for CD166/ALCAM (**a** to **g**) and IGF1-R (**h** to **n**). Positive staining was observed in glandular epithelium (brown and arrows) and counterstained with Hematoxylin (blue nuclei). (**a** and **h**) Human tonsil tissue was used as the positive control. (**b** and **i**) Human endometrium with no primary antibody served as the negative control. (**c** and **j**) Ovulatory women. (**d** and **k**) untreated anovulatory infertile women. (**e** and **l**) CC-treated anovulatory infertile women. (**f** and **m**) rFSH-treated women. (**g**) Optical density analysis of CD166/ALCAM in the glandular epithelium a significant higher expression (*P < 0.05) in CC-treated group compared with ovulatory and untreated infertile women. (**n**) Optical density analysis for IGF1-R showed a significantly lower expression (*P < 0.05) in glandular epithelium of rFSH-treated women compared with the other groups. GE = glandular epithelium; LE = luminal epithelium; S = stroma; OP = ovulatory women; CC = anovulatory infertile patients treated with clomiphene citrate; rFSH = anovulatory infertile patients treated with the recombinant follicle stimulating hormone; UI = anovulatory untreated patients. (Bar = 100 μm)

The expression of CD166/ALCAM was significantly higher in the CC-treated group compared with ovulatory and untreated infertile women (Fig. 5). CD166/ALCAM distribution was observed in the cytoplasm and basolateral membrane of glandular epithelial cells in CC- and rFSH-treated patients, while in ovulatory women there was a predominant distribution only in the basolateral membrane. Moreover in untreated infertile women no staining was predominant, and in contrast to treated patients a cytoplasmic staining was observed (Fig. 2g).

The expression of IGF-1R was also observed in the cytoplasm/membrane in glandular epithelium, with a significantly lower expression in the rFSH-treated group compared with all other groups (Fig. 5). IGF-1R expression was observed mainly in cytoplasm and basolateral membrane in the ovulatory group, whereas in the other groups, cytoplasmic distribution was predominant (Fig. 2h).

4) No differences were detected in the expression of endometrial stromal receptivity markers

To determine the expression of stromal receptivity markers in mid-secretory endometrium, immunohistochemistry assays were performed to evaluate the following markers: VEGF, TGF-β, CD34, CD44 and CD31/PCAM-1. Positive staining was observed for all markers preferentially in the cytoplasm or membrane of the stromal cells, with no significant differences between groups (Additional file 2: Figure S2 and Additional file 3: Figure S3). Given that no differences were observed between groups, we summarized the quantification and cellular distribution of these markers expression in Fig. 3.

**Fig. 6:**
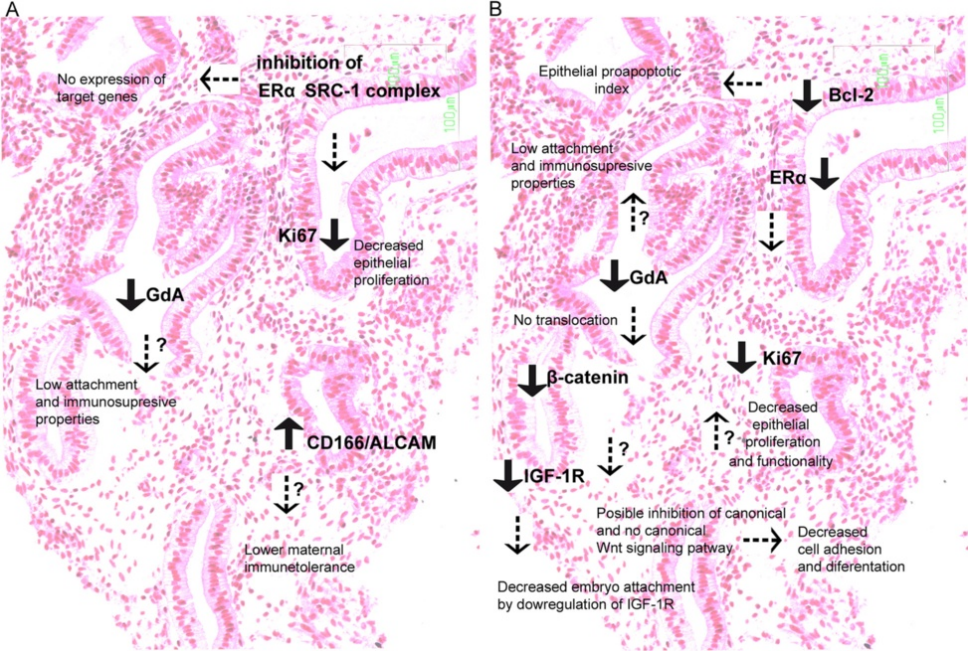
Summary of the main molecular changes and their interactions in infertile women with HPD, and differences between treatments: (**a**) CC treated women and (**b**) rFSH treated women. Black arrows indicate the changes in expression of different markers. Dashed arrows indicate the possible effects of these protein markers alterations

To improve the understanding of the overall results of this study in a physiological context, Fig. 6 summarizes the main molecular endometrial alterations observed after treatments with ovulation inducing agents and their possible interactions.

## Discussion

The medical treatment of first choice in HPD, to restore the synchrony of ovarian follicular development is the administration of ovulation-inducing agents, such as CC or rFSH [22,23]. However, the effect of these treatments on endometrial receptivity is under extensive investigation because of low rates of pregnancy observed after treatments.

In a previous study, we demonstrated that CC and rFSH treatments modified the expression of endometrial functionality markers related with proliferation and cell death in mid-secretory endometrium of HPD infertile women [15], indicating that molecular evaluation of the endometrial function is reliably to characterize the receptive phase [24–26].

In the present study the expression and distribution analysis of different glandular epithelial and stromal markers of receptivity was performed in women with HPD and after treatment with CC and rFSH. The molecules reported here included epithelial GdA, LIF, SCF, C-Kit, E-cadherin, beta-catenin, IGF-1R and CD166/ALCAM and stromal VEGF, TGF-β, CD34, CD31/PCAM-1 and CD44, markers, and were selected by two criteria: the involvement in the epithelial function during the implantation window such as epithelial adhesion, modulators of cell invasion and growth factors as reported previously and their role in first steps of decidualization process of stromal cells.

It has been demonstrated that GdA, LIF, c-Kit and SCF have a role in endometrial receptivity by modulating different physiological aspects of trophoblast invasion, the feto-maternal defense and cell adhesion [7,27,28]. Previous studies have reported a lower expression of GdA, progesterone receptor B and LIF in the mid-secretory endometrium of infertile women [29]. Here, we found that GdA expression was significantly lower in the glandular epithelial cells of infertile patients treated with CC or rFSH compared with ovulatory women. Recent studies indicates that serum GdA/IGFBP-1 ratio was higher in women who achieved pregnancy compared to those who did not [30], and in the uterine fluid of infertile women exhibited lower concentration of GdA as compared with fertile women [31]. Our results concur with this idea and suggest a detrimental effect of CC or rFSH treatments on endometrial GdA expression.

It is well known that LIF plays a similar role to GdA during implantation [32]. For example, in a previous study it was demonstrated an interaction between embryo and endometrium mediated by cell adhesion molecules. They also indentified cytokine-cytokine receptor interactions such as LIF, osteopontin, apolipoprotein D, fibroblast growth factor 7 between other molecules [33]. However, in our study, the endometrial expression of LIF was similar in all groups. This is in agreement with a previous study where did not find differences between fertile and infertile women [34]. This indicates that LIF could play a different role during this endometrial phase or is under complex regulation, where CC or rFSH treatment did not modify its expression.

During the implantation development, SCF exerts a paracrine action by stimulating the trophoblast through its c-Kit receptor. It was reported that SCF and c-Kit were highly expressed in implanted human blastocysts [35], suggesting a major role in this process. However, in our study we did not find any significant differences in endometrial SCF and c-Kit levels between groups. This suggests, that SCF and c-Kit could not be involved in the receptivity of the endometrial tissue.

During the receptive phase, it has been demonstrated that the endometrial expression of cell adhesion molecules (CAMs) is sharply increased and contributes to blastocyst attachment to uterine epithelium [36]. In the present study a significantly lower expression of beta-catenin was observed in the glandular epithelium in the rFSH-treated group compared with ovulatory and CC-treated women. Contrarily, no significant differences were found in E-cadherin expression between groups. These findings are important due to the fact that beta-catenin has uterine function and participates in the regulation of endometrial epithelial differentiation during endometrial receptivity period [36,37]. Interestingly, a recent *in vitro* study demonstrated a link between GdA and the translocation of beta-catenin to promote cell adhesion and formation of adherent junctions through cytoskeletal reorganization in human endothelial cells [38]. This correlates well with our findings of a significant decrease in both GdA and beta-catenin expression of rFSH treatment. However, further studies are needed to determine the accurate mechanism of beta-catenin role in endometrial function after rFSH treatment.

It has been demonstrated that ALCAM is expressed in the endometrium and blastocysts during implantation. Interestingly, we found a significantly higher expression of this protein in the glandular epithelium of the CC-treated group compared to ovulatory and untreated infertile women. This finding is interesting because it is also known that CD166/ALCAM, activate an immunological response through the binding of T lymphocytes with their CD6 receptor [39,40]. This suggests that CC treatment could participate in the activation of endometrial immunological response. However, more investigation is required to further define the molecular mechanism of CD166/ALCAM action.

It has been shown that IGF-1R, IGFBP-1 and its receptor may also play an important role during embryo implantation by facilitating adhesion processes [41], and extravillous trophoblast cell migration [42]. These factors are regulated during the secretory phase mainly by progesterone [43]. In our study, IGF-1R expression in glandular epithelial cells showed a significant diminution of this receptor in rFSH-treated group compared to other groups, indicating that the lower expression observed may have an influence in endometrial receptivity and with the low rate of pregnancy. This idea is supported by a recent work that demonstrated the over expression of miR-145 suppressed embryo epithelial communication by the modulation of the expression of maternal IGF-1R in endometrium which leads to implantation failure [44].

On the other hand, we did not observe differences in the expression of stromal receptivity markers such as VEGF, which promote decidualization and vascularization. This molecule, together with CD31, CD34, CD44 regulates vascular permeability, endothelial cell proliferation and cell migration, which are involved in endometrial receptivity [45]. However, few or any studies have explored the role of endometrial VEGF expression in infertile patients under treatments [46,47]. Moreover, compared with fertile women, patients with unexplained infertility have lower levels of VEGF in the uterine fluid [48]. Contrarily, in the present study no significant differences in VEGF expression were found between groups. However, in this regard, reports remains controversial and deserve further investigation.

The CD34, and CD31/PCAM-1 markers are transmembrane proteins related with vascular development and adhesion in endothelial cells of the endometrial stroma [49,50]. CD44 is expressed in the stroma, vessels and glandular epithelium, and interacts with proteoglycan, and also proposed to function as a mediator of embryo-endometrial interaction [51]. However, no significant differences between groups for CD44, CD34, or CD31/PCAM-1 were detected.

It has been shown that TGF-β modulates maternal immunotolerance during implantation. Besides, *in vitro,* this cytokine regulates several molecules related to implantation, such as VEGF, MMP-9, IGFBP-1 and LIF [52]. In our study, we did not find a significant difference in TGF-β expression between groups this may be due that TGF-β has a timing expression, or it is not related with receptivity window during secretory phase. The results related to cell marker distribution could be important to explain the alteration at cellular level in endometrial receptivity after stimulation treatments.

As we summarized in Fig. 6, the major findings of this study were that in both ovarian stimulation treatments, several markers expression were differentially modified in the epithelial cells and the main alterations were observed in the expression of cell adhesion, cell proliferation, cell signaling and growth factors that could be related with lower embryo implantation success. However, the accurate role of each marker during implantation and pregnancy should be well established and deserved more experimental research. Overall findings of this study could be relevant for the interpretation of treatments molecular effects in terms of clinical applications that allow to increase the success of pregnancy.

## Conclusion

The overall results indicated that treatments with ovulation inducing agents modify the expression and distribution of epithelial but not of stromal receptivity markers in the endometrium during the mid-secretory phase. Thus, suggesting an important role of epithelial cells during blastocyst implantation. The insights of this study may contribute in understanding of molecular interactions and endometrial function in the receptive phase, which should be take in account to improve pregnancy rates after treatments with ovulation inducing agents on infertile women.

## Additional files

Additional file 1: Figure S1. Immunohistochemistry for SCF (A to G) and c-Kit (H to N). Positive staining was observed in glandular epithelium and stroma (brown and arrows), which was counterstained with Hematoxylin (blue nuclei). (A and H) Human tonsil tissue was used as the positive control. (B and I) Human endometrium with no primary antibody served as the negative control. (C and J) Ovulatory women. (D and K) Untreated anovulatory infertile women. (E and L) CC-treated anovulatory infertile women. (F and M) rFSH-treated women. (G and N) Optical density analysis of SCF and c-Kit showed no significant differences in the expression of the markers between groups (*P<0.05). GE= glandular epithelium; LE= luminal epithelium; S= stroma; OP= ovulatory women; CC= anovulatory infertile patients treated with clomiphene citrate; rFSH= anovulatory infertile patients treated with the recombinant follicle stimulating hormone; UI= anovulatory untreated patients. (Bar=100 μm). Additional file 2: Figure S2. Endometrial expression of stromal receptivity markers. Immunohistochemistry for TGF-β (A to G) and VEGF (H to N). Positive staining was observed in glandular epithelium and stroma (brown and arrows), which was counterstained with Hematoxylin (blue nuclei). (A and H) Human colon cancer tissue was used as the positive control. (B and I) Human endometrium with no primary antibody served as the negative control. (C and J) Ovulatory women. (D and K) Untreated anovulatory infertile women. (E and L) CC-treated anovulatory infertile women. (F and M) rFSH-treated women. (G and N) Optical density analysis of TGF-β and VEGF showed no significant differences in the expression of the markers between groups (*P<0.05). GE= glandular epithelium; LE= luminal epithelium; S= stroma; OP= ovulatory women; CC= anovulatory infertile patients treated with clomiphene citrate; rFSH= anovulatory infertile patients treated with the recombinant follicle stimulating hormone; UI= anovulatory untreated patients. (Bar=100 μm). Additional file 3: Figure S3. Endometrial expression of stromal receptivity markers. Immunohistochemistry for CD34 (A to G), CD44 (H to N) and (O to U) CD31/PCAM-1. Positive staining was observed in glandular epithelium and stroma (brown and arrows), which was counterstained with Hematoxylin (blue nuclei). (A, H and O) Human tonsil tissue was used as the positive control. (B, I and P) Human endometrium with no primary antibody served as the negative control. (C, J and Q) Ovulatory women. (D, K and R) Untreated anovulatory infertile women. (E, L and S) CC-treated anovulatory infertile women. (F, M and T) rFSH-treated women. (G, N and U) Optical density analysis of CD34 and CD44 and CD31 showed no significant differences in the expression of the markers between groups (*P<0.05). GE= glandular epithelium; LE= luminal epithelium; S= stroma; OP= ovulatory women; CC= anovulatory infertile patients treated with clomiphene citrate; rFSH= anovulatory infertile patients treated with the recombinant follicle stimulating hormone; UI= anovulatory untreated patients. (Bar=100 μm).
