# Supplementary figures and images for: Changes in receptivity epithelial cell markers of endometrium after ovarian stimulation treatments: its role during implantation window
Source: Reprod Health. 2015 May 17;12:45. doi: 10.1186/s12978-015-0034-7 (PMC4443517; doi:10.1186/s12978-015-0034-7)

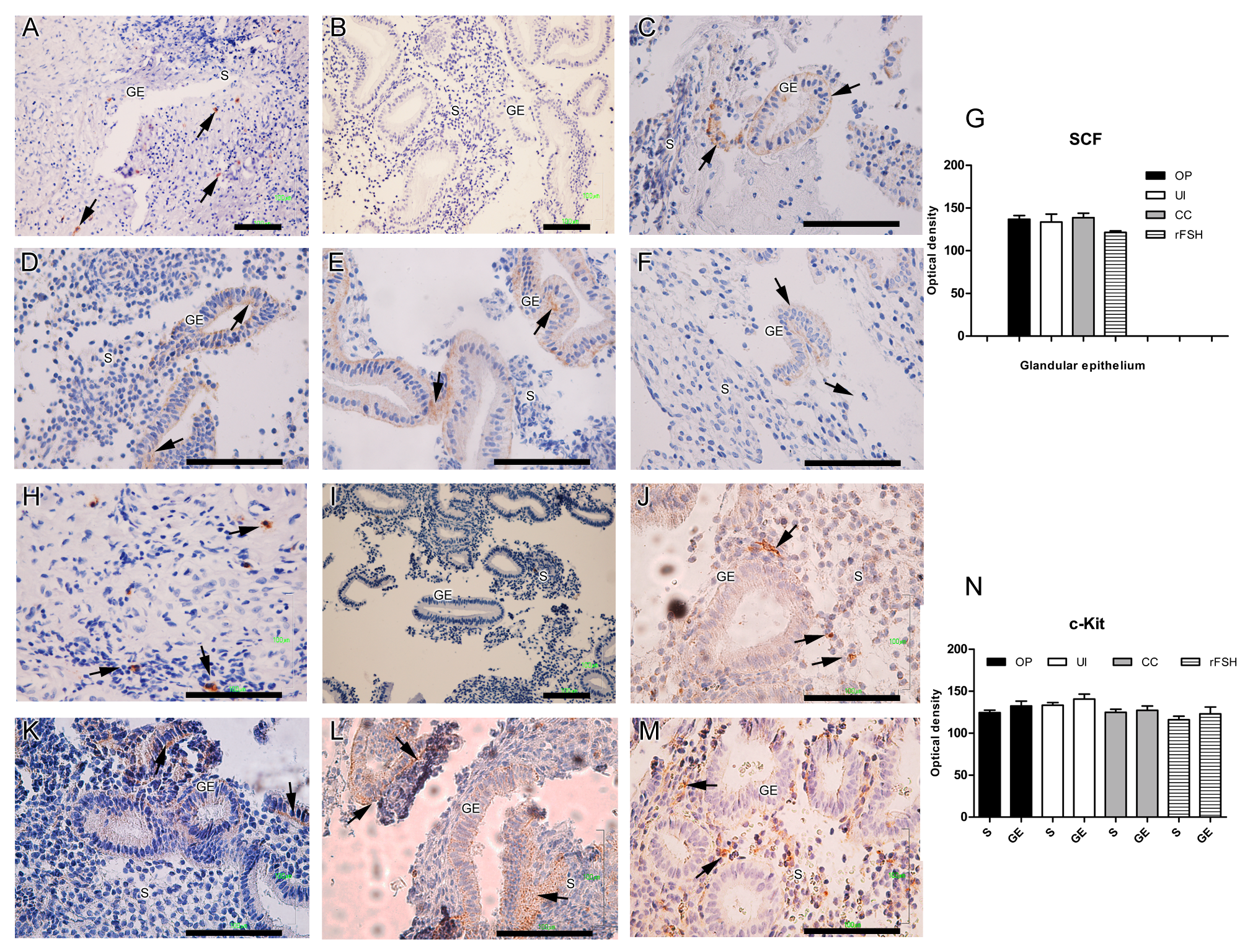

Supplement: Additional file 1: Figure S1. — Immunohistochemistry for SCF (A to G) and c-Kit (H to N). Positive staining was observed in glandular epithelium and stroma (brown and arrows), which was counterstained with Hematoxylin (blue nuclei). (A and H) Human tonsil tissue was used as the positive control. (B and I) Human endometrium with no primary antibody served as the negative control. (C and J) Ovulatory women. (D and K) Untreated anovulatory infertile women. (E and L) CC-treated anovulatory infertile women. (F and M) rFSH-treated women. (G and N) Optical density analysis of SCF and c-Kit showed no significant differences in the expression of the markers between groups (*P<0.05). GE= glandular epithelium; LE= luminal epithelium; S= stroma; OP= ovulatory women; CC= anovulatory infertile patients treated with clomiphene citrate; rFSH= anovulatory infertile patients treated with the recombinant follicle stimulating hormone; UI= anovulatory untreated patients. (Bar=100 μm). [file 12978_2015_34_MOESM1_ESM.tiff]

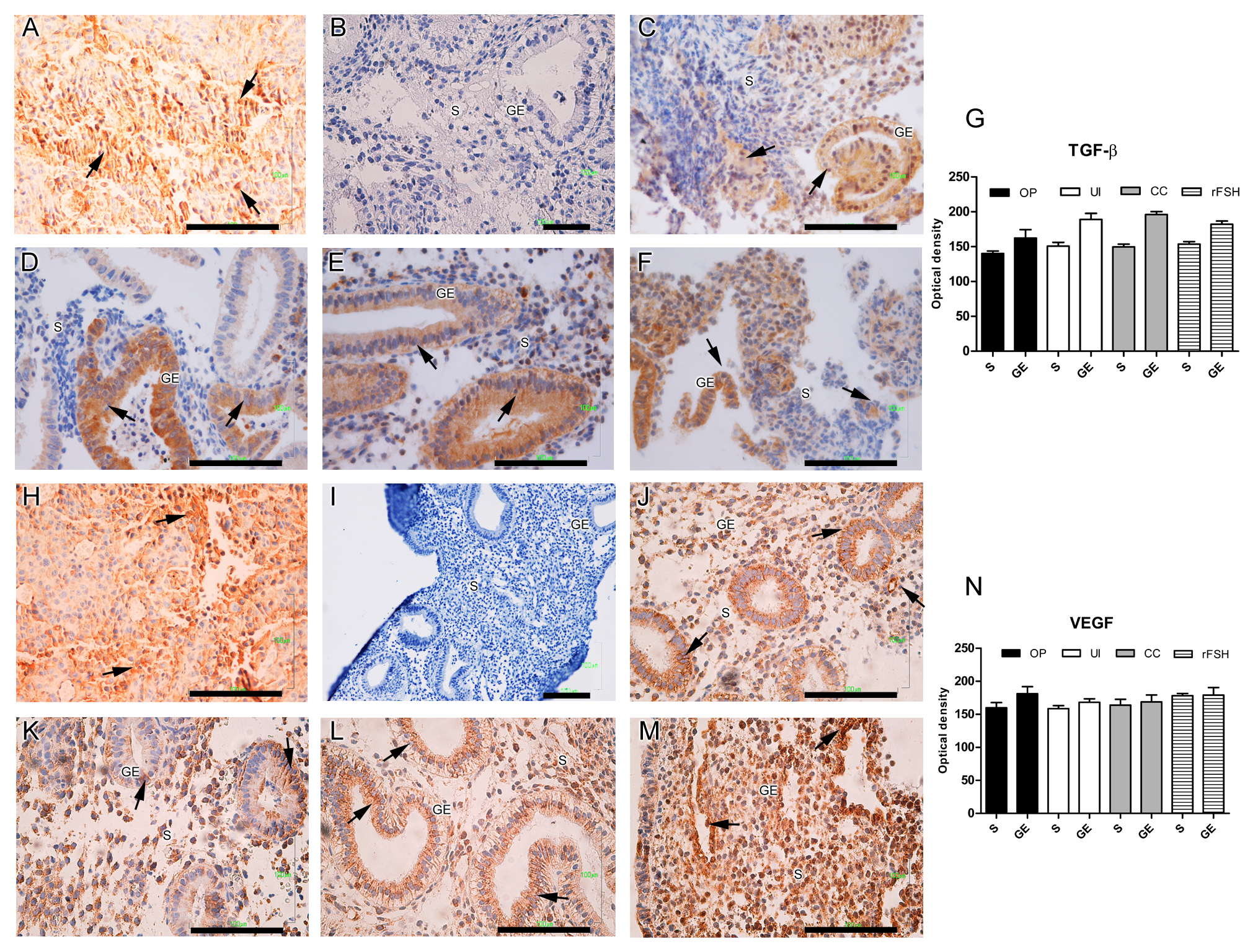

Supplement: Additional file 2: Figure S2. — Endometrial expression of stromal receptivity markers. Immunohistochemistry for TGF-β (A to G) and VEGF (H to N). Positive staining was observed in glandular epithelium and stroma (brown and arrows), which was counterstained with Hematoxylin (blue nuclei). (A and H) Human colon cancer tissue was used as the positive control. (B and I) Human endometrium with no primary antibody served as the negative control. (C and J) Ovulatory women. (D and K) Untreated anovulatory infertile women. (E and L) CC-treated anovulatory infertile women. (F and M) rFSH-treated women. (G and N) Optical density analysis of TGF-β and VEGF showed no significant differences in the expression of the markers between groups (*P<0.05). GE= glandular epithelium; LE= luminal epithelium; S= stroma; OP= ovulatory women; CC= anovulatory infertile patients treated with clomiphene citrate; rFSH= anovulatory infertile patients treated with the recombinant follicle stimulating hormone; UI= anovulatory untreated patients. (Bar=100 μm). [file 12978_2015_34_MOESM2_ESM.tiff]

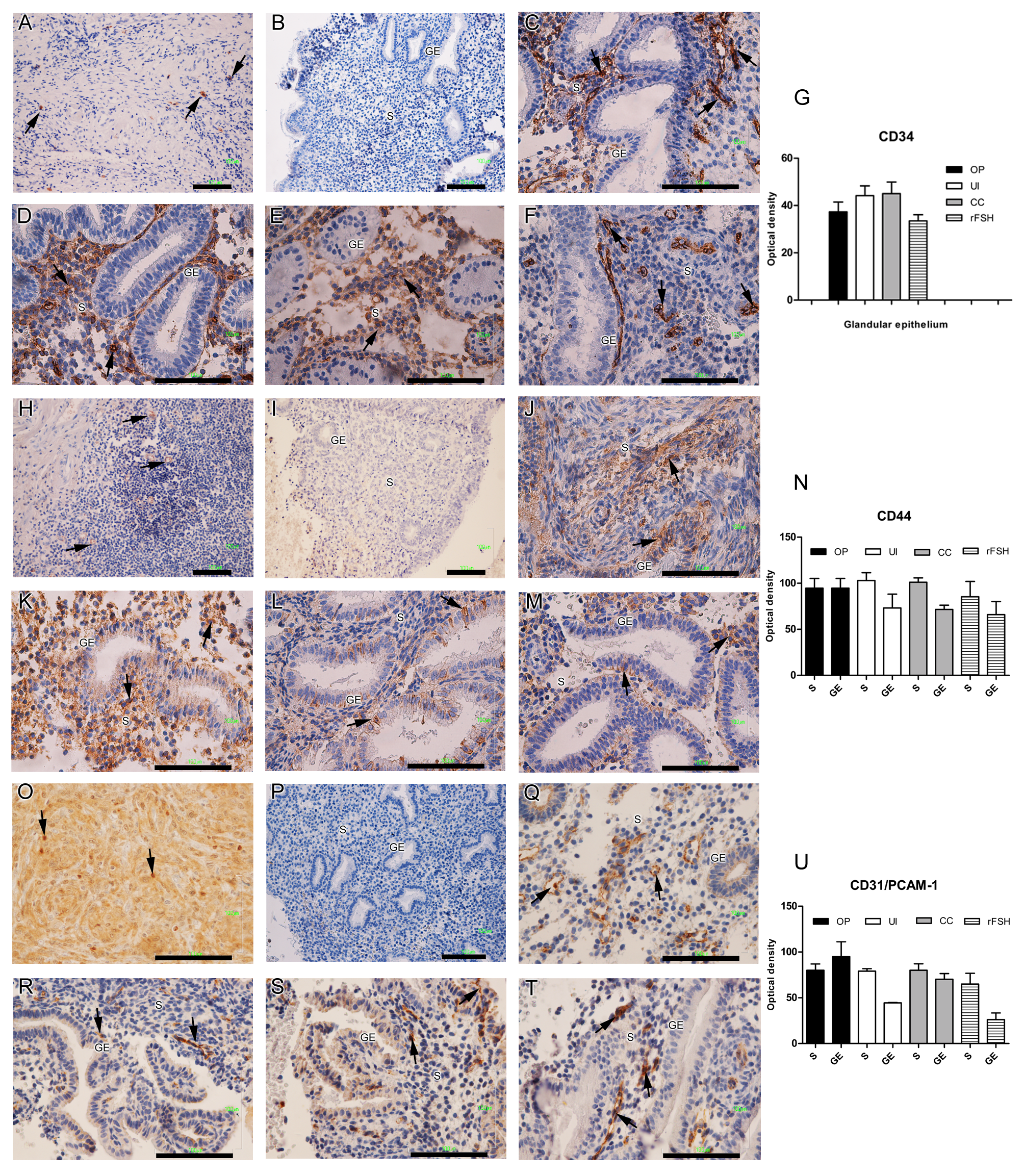

Supplement: Additional file 3: Figure S3. — Endometrial expression of stromal receptivity markers. Immunohistochemistry for CD34 (A to G), CD44 (H to N) and (O to U) CD31/PCAM-1. Positive staining was observed in glandular epithelium and stroma (brown and arrows), which was counterstained with Hematoxylin (blue nuclei). (A, H and O) Human tonsil tissue was used as the positive control. (B, I and P) Human endometrium with no primary antibody served as the negative control. (C, J and Q) Ovulatory women. (D, K and R) Untreated anovulatory infertile women. (E, L and S) CC-treated anovulatory infertile women. (F, M and T) rFSH-treated women. (G, N and U) Optical density analysis of CD34 and CD44 and CD31 showed no significant differences in the expression of the markers between groups (*P<0.05). GE= glandular epithelium; LE= luminal epithelium; S= stroma; OP= ovulatory women; CC= anovulatory infertile patients treated with clomiphene citrate; rFSH= anovulatory infertile patients treated with the recombinant follicle stimulating hormone; UI= anovulatory untreated patients. (Bar=100 μm). [file 12978_2015_34_MOESM3_ESM.tiff]
